# Morphine compared to placebo for procedural pain in preterm infants: safety, efficacy and equipoise

**DOI:** 10.1038/s41372-019-0476-9

**Published:** 2019-08-27

**Authors:** Omri David Soffer, Laura Cornelissen, Christy Cummings, Charles Berde

**Affiliations:** 1 Division of Newborn Medicine, Boston Children’s Hospital, Boston, MA USA; 2 Division of Pain Medicine, Department of Anesthesiology, Critical Care and Pain Medicine, Boston Children’s Hospital, Boston, MA USA

**Keywords:** Neurophysiology, Medical ethics, Ethics

**Manuscript citation:** C. Hartley, F. Moultrie, A. Hoskin, G. Green, V. Monk, J. L. Bell, et al. Analgesic efficacy and safety of morphine in the Procedural Pain in Premature Infants (Poppi) study: randomised placebo-controlled trial. Lancet. 2018;392:2595–2605

## Type of investigation

Treatment.

## Question

Among nonventilated preterm infants, who are less than 32 weeks of gestation or with birth weight less than 1501 g, undergoing an eye examination to detect retinopathy of prematurity (ROP) or heel lancing, will a 0.1 mg/kg single oral dose of morphine, compared to a placebo solution, be safe, and provide analgesic efficacy demonstrated by Premature Infant Pain Profile-Revised scoring system (PIPP-R) and noxious-evoked brain activity (nEBA) while maintaining physiological stability?

## Methods

**Design:** Randomized, double-blinded, placebo-controlled clinical trial.

**Follow-up period:** 24 h after the eye exam and heel lancing.

**Setting:** Single-center, academically affiliated neonatal intensive care unit (NICU).

**Patients:** (1) Inclusion criteria: Nonventilated preterm infant born at less than 32 weeks’ gestation OR with a birth weight of less than 1501 g and aged 34–42 weeks’ gestation (postmenstrual age) AND requiring eye exam for ROP screening or a heel lance. (2) Exclusion criteria: Infants with grade 3 or 4 intraventricular hemorrhage, short bowel syndrome, nil per os status, congenital/genetic conditions with probable neurodevelopmental manifestations, sedative or opiate exposure 24 and 72 h prior to ROP examination or heel lancing, respectively, documented opiate sensitivity, history of maternal opiate exposure either in utero or while breastfeeding, mechanical ventilation at the time of the trial.

**Intervention:** Preterm infants were randomized to a morphine group (received a single oral dose of morphine sulfate 0.1 mg/kg) or control group (received oral placebo solution similar in color, odor, and volume). Solutions were given approximately 1 h prior to ROP exam and heel lancing. Investigators and clinical team were blinded.

### Outcomes

- **Co-primary outcomes:** Premature Infant Pain Profile-Revised (PIPP-R score after ROP exam); magnitude of nEBA after heel lancing.
- **Secondary outcomes:** limb reflex withdrawal, PIPP-R scores after heel lancing, clinical stability determined by episodes of apnea, bradycardia, tachycardia, desaturation and escalation in respiratory support during the 6 and 24 h after the clinical procedures, drug safety determined by the incidence of apnea requiring noninvasive positive pressure ventilation and hypotension requiring vasopressors 24 h after administering morphine or placebo.

**Analysis and sample size:** 132 infants were needed to detect a 2-point reduction in PIPP-R score, defined as a clinically significant reduction, from a conservative postretinopathy of prematurity screening mean PIPP-R score of 8.3 (SD 3.5) from a previous study, with power of 90% and alpha of 0.05 (two-tailed). Additionally, a 40% relative risk reduction in noxious-evoked brain activity was defined as clinically significant with 90% power and 5% significance. The investigators aimed for a sample size of 156 infants to account for multiple births and anticipated 10% loss of follow-up. Values were reported in mean and median values. PIPP-R scores were compared between the two groups using a *t* test. The magnitudes of noxious-evoked brain activity were compared between the groups using a Wilcoxon rank-sum test.

**Patient follow-up:** Infants were followed for 24 h after ROP examination and heel lancing after receiving morphine or placebo oral solutions. The trial was stopped early at 19% enrollment (30 infants) by the study’s Data Monitoring Committee 1 year after enrollment had commenced using predefined stopping criteria due to a concern for patient safety (profound respiratory adverse effects) without evidence of analgesic efficacy.

## Main results

The baseline characteristics and demographics of the study participants were balanced on some aspects (e.g. gestational and postnatal ages, birth weight) and unbalanced in others as expected in a small sample size (15 patients in each group). For example: the number of infants who required ventilator support (6 in the morphine group and 12 in the placebo group) and the number of infants who were previously exposed to morphine (4 in the morphine group and 7 in the placebo group).

The efficacy outcomes showing no difference between morphine and placebo group is shown in Table 1. The safety outcomes, reported as median of the standarized difference in number of episodes before and after intervention,   have shown significant higher episodes in desaturation 0.66 (95% CI: 0.36–1.00, *p* = 0.0007) and bradycardia 0.33 (CI: 0.00–0.98, *p* = 0.07) in the morphine group at 6 h as well as 24 h for desaturation 0.33 (CI: 0.03–0.75, *p* = 0.019) and bradycardia 0.43 (CI: 0.00–1.00, *p* = 0.019).

**Table. 1:** Efficacy outcomes compared between the morphine group and the placebo group

Efficacy outcomes	Morphine group (*n* = 15)	Placebo group (*n* = 15)	Mean/median difference	*p* value
PIPP-R score after ROP exam (mean ± SD)	11.1 ± 3.2	10.5 ± 3.4	0.5	0.66
nEBA after heel lancing (median, IQR)	0.99 (0.4–1.56)	0.75 (0.33–1.22)	0.25	0.25
PIPP-R score after heel lancing (mean ± SD)	7.9 ± 3.4	8.5 ± 3.9	−0.6	0.66
Reflex withdrawal magnitude after heel lancing (median, IQR)	24.8 (19.7–44.8)	12.4 (6.1–46.3)	8.9	0.48

*PIPP-R* Premature Infant Pain Profile-Revised scoring system, *ROP* retinopathy of prematurity, *nEBA* noxious-evoked brain activity

## Study conclusion

Oral morphine administration to nonventilated premature infants is not recommended due to its potential harm and lack of analgesic effect at the proposed dosage (0.1 mg/kg).

## Commentary

Previous clinical trials have yet to demonstrate effective, minimal risk analgesic management in neonates undergoing ROP eye examination [1]. Accurate pain assessment in infants is challenging for clinicians since infants cannot provide self-report. Indirect measures of pain assessment such as heart rate, oxygen saturation, and body movement are used as surrogate measures [2]. These indirect measures are mediated at the spinal and subcortical level, and may not accurately reflect pain perception at the level of the cortex [3]. To overcome this barrier, the study investigators used an innovative study design incorporating multimodal measures of pain assessment including EEG-based indices in combination with a traditional pain score (PIPP-R). The study hypothesis was that analgesic efficacy, measured through a reduction of cortical nEBA and PIPP-R scores, would be seen in the morphine arm, compared to the placebo arm.

The study showed that reduction in PIPP-R score, noxious-evoked brain activity magnitude and EMG reflex withdrawal magnitude did not differ significantly between the morphine and the placebo group. However, the trial had to be stopped early according to predefined stopping criteria due to clinical instability of patients in the morphine group (profound respiratory adverse effects).

No other trials to date have been conducted to specifically assess the analgesic efficacy and safety of morphine administration for ROP examination. Several randomized controlled trials have evaluated the analgesic efficacy of acetaminophen for ROP exam to demonstrate a possible reduction in Premature Infant Pain Profile (PIPP) score during the examination. However, those trials demonstrated either modest reduction in PIPP score after acetaminophen was given compared to sterile water or no significant reduction in PIPP score followed by oral sucrose solution [4, 5]. Moreover, those trials only utilized the PIPP scoring system to assess analgesic effect, ultimately relying on behavioral and autonomic activity. There is one ongoing study that may provide more informative pain assessment in newborns using different modalities to assess infants’ nociceptive and physiologic responses to noxious stimuli [6].

The study findings call into question the *safety* of morphine, at the dose used, for acute procedural pain relief in nonventilated preterm infants, given the notable increase in adverse effects. An enteral dose of 0.1 mg/kg of morphine may have been a high initial dose, especially given a significant number of the study participants were morphine naïve (73%), potentially leading to increased apnea, bradycardia, and desaturation episodes in this group. Therefore, considering the clinical instability of many of the infants receiving enteral morphine, a lower dose may have led to a more preferable safety outcome, although unlikely to have demonstrated analgesic efficacy. The trial was left underpowered, resulting in an inability to make confident conclusions regarding the analgesic efficacy of morphine in this population.

A recent meta-analysis of randomized trials compared topical ophthalmic anesthetics to various pain-relieving interventions for ROP examination. Despite evidence that multisensory pain treatments (i.e. oral dextrose solution, expressed breast milk, rocking, singing, nonnutritive sucking, swaddling, and acetaminophen) may reduce infants’ pain scores to varying degrees, no specific treatment necessarily provided clinically meaningful pain relief. The majority of infants in the trials had a mean PIPP score higher than 12 (i.e. moderate to severe pain) [1]. Therefore, safe and effective analgesics for nonventilated preterm infants undergoing ROP exam is still needed.

In conclusion, this trial is notable for its novel use of innovative pain assessment tools (EEG and EMG), in addition to the more subjective PIPP-R score. Ongoing trials will hopefully provide more informative and objective pain assessment in newborns using different modalities to assess nociceptive and physiological activity, when PIPP-R scores or other behavioral-based pain assessments are not applicable such as in a state of heavy sedation or neuromuscular blockade. Brain-based assessment modalities such as EEG may also provide valuable information about pain in infants and guide optimal management.

## EBM lesson: Ethical challenges in conducting randomized controlled trials of analgesics in newborns

Conducting a randomized controlled trial of analgesics compared to placebo involving newborns has distinct ethical challenges. In clinical research, equipoise assumes genuine uncertainty regarding treatment superiority. When equipoise has been disrupted, the trial should then be stopped according to predetermined criteria and interim safety analysis, as in the POPPI Trial. The justification for placebo controls should be determined on the basis of the research question, and current standard of care. However, the lack of a clear standard of care for treatment of neonatal pain raises a valid concern in clinical trials; equipoise is further complicated when data are lacking about either treatment in a randomized clinical trial. The POPPI trial was designed as a standard versus experimental study (i.e. ophthalmic topical anesthetics and swaddling versus morphine administration). Given the lack of standard of care for these clinical situations, one therapeutic measure was compared with another; both are considered “off-label.” Without a standard treatment for pain control, then a placebo might be considered ethically acceptable. Using the POPPI trial as an example, if most providers did not use topical ophthalmic anesthetics, then it would be ethically permissible to test morphine against placebo. If a proven effective and safe means of pain control for a particular indication in a particular population exists, then a placebo-controlled trial would be ethically impermissible and new treatment must be tested against the efficacious treatment. Testing a new therapy as potential adjuvant to the “standard” therapy allows all subjects to receive the “standard” therapy, thus an ethically permissible approach. In the POPPI trial, this strategy was utilized where all the study participants were swaddled and received topical ophthalmic anesthetics [7, 8].

The American Academy of Pediatrics (AAP) has outlined conditions under which the use of placebos may be ethically acceptable: (1) lack of accepted therapy for the condition and the agent under study may modify the course of the disease process (in this case, procedural pain); (2) questionable efficacy of the commonly used therapy; (3) known adverse events for the commonly used therapy; (4) the placebo is used to identify undesirable side effects produced by adding a new treatment; and (5) the disease process is characterized by frequent, spontaneous exacerbations and remissions, without satisfactory therapy [9]. Considering procedural pain as the disease process in this case, all the outlined criteria above may be applicable to the POPPI trial. As pain is undeniably unfavorable, potentially effective and safer analgesic methods should be investigated.

In general, the efficacy and safety of an analgesic should be compared against the best current therapeutic methods. The use of placebo may be permissible in studies where no proven therapeutic intervention exists; in other words, a true state of equipoise [8]. Why then is a placebo control group acceptable if pain is ethically and morally unacceptable and analgesia is preferable; why not just treat the patient? The answer lies in the uncertainty of the benefits gained and risks involved in the proposed treatment, given the limitations of current assessment methods. Out of necessity, unapproved and understudied drugs are commonly used in neonatal care; going forward clinical research of drugs in newborns will be even more important, as well as input from the institutional board governing these studies [8].

In summary, the POPPI trial demonstrated various ethical challenges in designing clinical trials involving newborns, including justifying placeboes, defining equipoise and highlights the need for future studies in this area.
